# Artificial Neural Network-Based PID Parameter Estimation Using Black Kite Algorithm Hyperparameter Optimization for DC Motor Speed Control

**DOI:** 10.3390/biomimetics11040242

**Published:** 2026-04-03

**Authors:** Yılmaz Seryar Arıkuşu

**Affiliations:** Department of Electrical Engineering, Manisa Celal Bayar University, Manisa 45400, Türkiye; yilmaz.arikusu@cbu.edu.tr

**Keywords:** DC motor, Black Kite Algorithm (BKA), artificial neural network (ANN), PID controller tuning, metaheuristic algorithm, control design

## Abstract

This paper proposes a Black Kite Algorithm (BKA)-based hyperparameter optimization method for Artificial Neural Network (ANN) training, mitigating local minimum issues associated with conventional training techniques. The resulting BKA-ANN model is then employed to estimate PID controller parameters for DC motor speed regulation. A large-scale dataset of 100,000 samples was generated via MATLAB simulation, with reference speed and load torque stochastically varied, and optimal PID parameters determined by minimizing the ITAE criterion for each operating condition. The optimized controller was evaluated under various operating conditions including transient response, frequency domain analysis (phase margin and bandwidth), parametric robustness, and load disturbance suppression, along with control effort and energy consumption assessments. The proposed BKA-ANN approach was benchmarked against nine algorithms: hybrid atom search optimization-simulated annealing (hASO-SA), harris hawks optimization (HHO), Henry gas solubility optimization with opposition-based learning (OBL/HGSO), atom search optimization (ASO), henry gas solubility op-timization (HGSO), stochastic fractal search(SFS), grey wolf optimization (GWO), sine–cosine algorithm (SCA), and Standard ANN. Simulation results indicate that BKA-ANN achieves stable performance across all tested scenarios, with minimal oscillation and competitive settling time compared to the evaluated algorithms.

## 1. Introduction

Establishing suitable control parameters is a complicated process with several problems. Problems are generally resolved through trial and error with fundamental principles [1]. In modern industrial systems, accurate and dependable control of dynamic processes, like direct current (DC) motor speed regulation, is another major difficulty. This system frequently shows sensitivity to disturbances, time-dependent dynamics, and nonlinear behavior [2,3]. DC motors have been controlled using PID controllers, the most popular and straightforward kind of controller in modern industrial fields [4,5]. The PID controller parameters can be found using simple methods like Ziegler–Nichols [6] and Cohen–Coon [7]. In terms of setpoint tracking, Ziegler–Nichols tuning produces tuning that makes inconsistent results, especially when used with integrating systems [8].

The controller might not deliver the required performance at high speeds, even though a system might function effectively at low speeds. Optimizing the controller parameters is crucial to solving these issues. Optimization methods are often utilized for this purpose [1].

In the literature, the use of PID controllers in industrial settings has been the subject of numerous studies. PID-based control techniques have been used to control speed in manufacturing processes like assembly lines [9,10,11] and automated transportation systems [12]. In the automotive sector, Farag et al. proposed a PID-based control approach to improve the maneuverability of autonomous vehicles [13]. In the same way, PID controllers are frequently employed to manage conveyor belt speeds in order to preserve coordinated and effective material flow throughout industrial operations [14,15]. In robotics PID algorithms are widely used to precisely regulate the velocity of robotic platforms by controlling the motion of mobile robots by varying the speed of DC motors [16]. As highlighted by Carlucho et al. [17], the structural simplicity and effectiveness of PID controllers continue to make them one of the most widely used control strategies in both industrial automation and robotics [17].

Although its benefits are clear, there are several difficulties in designing and implementing PID controllers. In order to sustain satisfactory performance in the face of disturbances and changing operational conditions, control engineers must guarantee system stability [18,19,20,21]. Furthermore, a suitable trade-off between resilience and performance needs to be made. In industrial settings, a controller that performs well under ideal conditions but is not robust to disturbances may produce undesirable outcomes [22,23]. As a result, rigorous controller parameter tuning and design are necessary to provide dependable stability and peak performance [24,25].

The need for accurate speed control is further highlighted by the extensive usage of DC motors in industry. For this purpose, PID controllers have proven to be a reliable option; however, their efficacy heavily relies on suitable tuning strategies that address stability, robustness, and performance difficulties in real-world applications [3,26,27].

Intelligent control methods like fuzzy logic, machine learning, and metaheuristic optimization algorithms are used in a variety of DC motor control strategies. For regulating the speed of DC motors, a hybrid strategy combining the Ziegler–Nichols Quarter Decay method with the Fuzzy Logic Controller method is recommended [28]. Fuzzy logic-based control is a rule-based control widely used in nonlinear systems where conventional linear control methods are inadequate. It is especially preferred in BLDC motor control, especially when the mathematical modeling of the system is complex. An enhanced fuzzy PID controller is developed and proposed for regulating the acceleration and speed of a BLDC motor in contrast to traditional PID controllers [29]. An affordable electric two-wheeler achieved the desired speed more quickly by using fuzzy logic control to efficiently manage a BLDC motor [30]. Using the target function of Integral of the Square Error in terms of control energy and Integral of Time multiplied by the Square Error in terms of performance, another study proposed the PSO approach for DC motor speed control as a means of determining PID [31]. Metaheuristic optimization algorithm techniques were used to determine the PID controller’s parameters in BLDC motor control [32,33,34,35].

One of the intelligent control methods used in DC motor control is machine learning. Taş et al. used machine learning algorithms to adjust the PID parameters for BLDC motor control rather than using conventional techniques [36]. Real-time speed and range estimation is done using neural networks, and an MLP signal network allows for sensorless BLDC motor control [37]. Reinforcement learning (RL), a subfield of machine learning, has emerged as a promising technique that allows controllers to adapt in real time and dynamically optimize their performance [38,39,40].

Advanced control strategies such as Model Predictive Control (MPC) have also been widely investigated for DC motor applications. Several studies have demonstrated that MPC-based speed control can provide improved robustness, reduced overshoot, and faster dynamic response compared with conventional control techniques [41,42,43,44].

Machine learning techniques, particularly artificial neural networks (ANNs), have been widely adopted to model system dynamics and provide adaptive control signals. However, an ANN’s weight and bias configuration greatly affect its performance. Conventional training methods, such as backpropagation, often suffer from local minima entrapment and sluggish convergence. Although metaheuristic algorithms have been increasingly employed as global optimizers for ANN training, several limitations persist. Algorithms such as PSO and GWO are prone to premature convergence and reduced exploration efficiency in high-dimensional search spaces. Similarly, HHO and ASO may exhibit slow convergence when the fitness landscape is highly non-convex, as is the case in ANN weight optimization for PID tuning tasks. These shortcomings motivate the use of a more robust global optimizer. The Black Kite Algorithm (BKA) [45], a recently developed metaheuristic inspired by the foraging behavior of black kites, addresses these limitations by providing an effective balance between exploration and exploitation. By combining BKA with ANN, a controller can be developed that not only learns optimal control laws but also retains structural robustness under parameter variations and external load disturbances. From a biomimetic perspective, BKA exemplifies the engineering application of nature-inspired optimization, replicating the attacking and migration behaviors of black kites to navigate complex search spaces. This alignment with biomimetic principles makes BKA particularly well-suited for publication in the scope of Biomimetics, as it demonstrates how biological foraging strategies can be translated into effective computational tools for intelligent control system design.

Despite their widespread use, purely metaheuristic-based PID tuning methods exhibit well-documented theoretical limitations. GWO relies on a leadership hierarchy that reduces population diversity rapidly, leading to premature convergence in high-dimensional spaces [46]. SCA employs sinusoidal position updates that lack an effective escape mechanism once the search radius diminishes [47]. HHO’s rabbit-chasing mechanism becomes increasingly exploitative in later iterations, reducing search diversity [48]. ASO exhibits slow convergence when inter-agent forces become unbalanced in non-convex landscapes [49], while SFS may converge prematurely without adaptive step size control [50]. These shortcomings are evident in the comparative results: GWO and SCA showed extreme deviations of 1.51% and 2.31%, respectively, while SFS yielded the slowest settling time at 1.4709 s. Furthermore, while ANN offers strong generalization capability for mapping nonlinear system dynamics to optimal PID parameters, conventional backpropagation-based training suffers from local minima entrapment, as evidenced by the Standard ANN’s 11.70% overshoot and 0.7862 s settling time. The main goal is to evaluate the applicability of BKA as a hyperparameter optimizer for ANN-based PID tuning in DC motor speed regulation, addressing both limitations simultaneously. While hybrid metaheuristic-ANN approaches have been widely studied in intelligent control, the specific combination of BKA with ANN for DC motor PID parameter estimation has received limited attention in the literature, and this study aims to contribute to this area.

The core contributions of this research are summarized as follows:

- A dedicated PID parameter dataset was constructed by deriving the mathematical model of the DC motor and performing MATLAB-based simulations under stochastically varied operating conditions. The dataset comprises 100,000 samples with optimal PID gains determined via the ITAE criterion, providing a high-fidelity training foundation for the proposed neuro-controller.
- This study presents the application of a BKA-based hyperparameter optimization approach for ANN-driven PID tuning in DC motor speed regulation. The hyperparameter values for the ANN were determined using the BKA method, combining the adaptive learning capability of neural networks with the global search efficiency of metaheuristic optimization.
- The proposed controller was evaluated against nine algorithms, including GWO [46], SCA [47], HHO [48], ASO [49], SFS [50], hASO-SA [51], OBL/HGSO [52], HGSO [52], and the Standard ANN, demonstrating competitive tracking and stability performance.
- Through an in-depth performance envelope analysis, the BKA-ANN model demonstrates improved structural robustness, maintaining low-overshoot speed regulation under simultaneous parameter variations in armature resistance and torque constant. The proposed approach shows favorable performance compared to HHO [48], and ASO [49], OBL/HGSO [52] under external load torque disturbances.
- The proposed approach was evaluated through comprehensive transient and frequency–domain analyses, including rise time, settling time, overshoot and control effort and overall energy consumption analysis. The results indicate that BKA-ANN achieves the lowest rise time and settling time among the evaluated algorithms. Additionally, the proposed approach maintains lower control effort and energy consumption compared to OBL/HGSO and ASO.

The remainder of this article is organized as follows: Section 2 details the Materials and Methods, including the mathematical modeling of the DC motor and the BKA-ANN optimization procedure. Section 3 presents the Results and Discussion, offering an exhaustive comparative analysis under nominal conditions, parameter uncertainties, and load disturbances. Finally, Section 4 provides the Conclusion of the study and outlines potential future research directions.

## 2. Materials and Methods

In this study, a comprehensive methodological framework is developed for DC motor speed control. An Artificial Neural Network (ANN)-based intelligent tuning mechanism is integrated with a traditional Proportional-Integral-Derivative (PID) controller in the proposed method. The BKA was employed to tune the structural hyperparameters and weights of the ANN to ensure excellent performance and flexibility across various operating conditions. The MATLAB/Simulink (R2022b) environment was used for all mathematical modeling, control system design, data generation, and performance evaluation experiments. The following subsections detail the mathematical model of the system, the proposed optimization strategy, and the dataset generation process.

### 2.1. Mathematical Modeling of the DC Motor

The dynamic behavior of the motor is described by the following differential equations governing the electrical and mechanical components:

(1) $$V_{a}(t)=\;R_{a}\;i_{a}(t)+L_{a}\frac{d\;i_{a}(t)}{dt}+E_{b}(t)$$

(2) $$T_{m}(t)=J\frac{d\omega (t)}{dt}+B\omega (t)+T_{L}$$

The following assumptions are adopted in deriving the mathematical model: (i) the magnetic circuit is assumed to be linear and magnetic saturation is neglected; (ii) the back-EMF is proportional to the angular velocity; (iii) the viscous friction coefficient is assumed constant; and (iv) the torque is treated as an external disturbance input. Under these assumptions, applying the Laplace transform to Equations (1) and (2) with zero initial conditions yields:

(3) $$E_{b}\left(s\right)=K_{b}\Omega \left(s\right)$$

(4) $$E_{a}\left(s\right)=\left(L_{a}s+R_{a}\right)I_{a}\left(s\right)+E_{b}\left(s\right)$$

(5) $$T\left(s\right)=\left(Js+B\right)\Omega \left(s\right)=K_{t}I_{a}\left(s\right)$$

The transfer function G(s), relating the input voltage to the output speed, is derived as:

(6) $$G\left(s\right)=\frac{W\left(s\right)}{V_{a}}=\frac{K_{t}}{{(L}_{a}s+R_{a})\left(Js+B\right)+K_{t}K_{b}}$$

where Table 1 shows the DC motor parameters and values.

### 2.2. PID Control Structure

A Proportional-Integral-Derivative (PID) controller is employed to regulate the motor speed. The control law in the time domain is formulated as:

(7) $$u\left.\left(t\right.\right)=K_{p}e(t)+K_{i}\int_{0}^{t}e(t)dt+K_{d}\frac{de(t)}{dt}$$

where $u\left(t\right)$ denotes the control signal $K_{p}$ is the proportional gain, $K_{i}$ is the integral gain and $K_{d}$ is the derivative gain and $e\left(t\right)$ represent the error signal that defined as the difference between the reference speed $\omega_{ref}$ and the actual motor speed ($\omega_{act}$) as the following term

(8) $$e\left.\left(t\right.\right)=\omega_{ref}-\omega_{act}$$

### 2.3. Dataset Generation for DC Motor Controller

**Figure 1:**
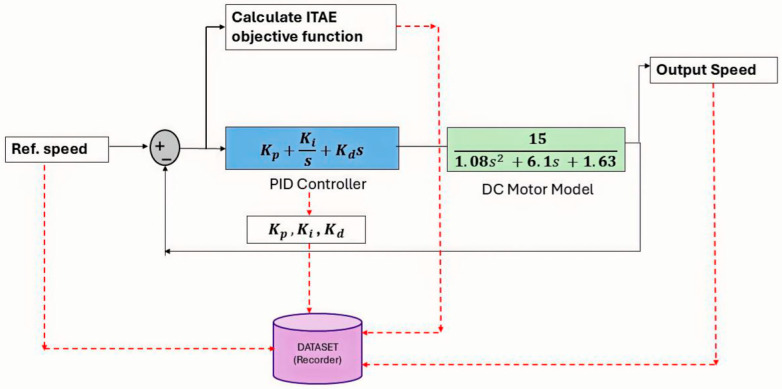
Dataset of the DC motor controller.

A large-scale dataset with 100,000 samples was created to guarantee that the proposed ANN delivers high precision in transient response management. The schematic flow of this data generation framework is illustrated in Figure 1. The operating conditions were stochastically varied, with the Reference Speed $\omega_{ref}$ distributed in the range of $\left[0.9,\;1.1\right]$ rad/s and the Load Torque $\left(T_{L}\right)$ in $\left[0,\;1.0\right]$ N.m. For each specific scenario, the optimal PID parameters $\left(K_{p},K_{i},K_{d}\right)$ were determined by minimizing the Integral of Time-weighted Absolute Error (ITAE) criterion, which is mathematically formulated as:

(9) $$J_{ITAE}=\int_{0}^{T_{sim}}t\cdot \left|e\left(t\right)\right|\;dt$$

where t denotes the time, $T_{sim}$ represents the simulation duration. A thorough high-fidelity dataset that maps the changing system dynamics to the corresponding optimal control gains was produced by this optimization procedure, offering a solid basis for the neuro-controller’s training.

Stochastic sampling was preferred over uniform grid sampling to ensure that the dataset captures the full variability of system dynamics rather than being biased toward regularly spaced operating points. This approach prevents the ANN from overfitting to a structured input distribution and improves its generalization capability across the continuous operating space.

### 2.4. Black Kite Algorithm (BKA)

The Black Kite Algorithm (BKA) is a novel bio-inspired meta-heuristic optimization algorithm recently proposed by Wang et al. (2024) [45]. The algorithm mimics the distinct biological behaviors of the black kite, a raptor renowned for its quick flying and ability to hunt. Attacking (exploitation) and Migration (exploration) are the two main behavioral phases that control the BKA optimization process.

In this phase, the algorithm refines the candidate solutions locally. A random number $r\in \left[0,\;1\right]$ determines the specific movement strategy. If r > p (where typically *p* = 0.9), the kite circles the prey, corresponding to a local search around the current position. Otherwise $\left.\left(r\leq \;p\right.\right),$ it executes an attack maneuver, which helps in converging towards the optimal solution. The position update is formulated as:

(10) $$X_{i}(a+1)=\left\{\begin{array}{ll} X_{i}(a)+\;n\left(1+sin\left(r\right)\right)X_{i}(a), & if\;r\;>\;p\\ X_{i}(a)+\;n\left(2r-1\right)X_{i}(a) & otherwise \end{array}\right.$$

where *n* is time-varying coefficient that shrinks the search radius as iterations progress.

In migration phase, to avoid stagnation in local optima, BKA employs a migration strategy integrated with Cauchy mutation. The algorithm compares the fitness of the current individual ($F_{i}$) with a randomly selected individual ($F_{ri}$). If the current individual is fitter ($F_{i}<F_{ri}$), it moves towards the global leader ($X_{best}$) utilizing Cauchy-distributed random steps $\left(C\left(0,1\right)\right)$ to enhance global exploration capabilities. The mathematical model is shown as follows.

(11) $$X_{i}(a)=\left\{\begin{array}{ll} X_{i\left(a\right)}+C\left(0,1\right){(X}_{i}(a)-X_{best}), & if\;F_{i}<F_{ri}\\ X_{i\left(a\right)}+C\left(0,1\right){(X}_{i}(a)-m{(X}_{i}(a)) & otherwise \end{array}\right.$$

This integration of Cauchy mutation significantly improves the algorithm’s ability to escape local optima compared to standard random walks. The complete computational flow of the BKA is summarized in Table 2.

### 2.5. Artificial Neural Network (ANN) Architecture

**Figure 2:**
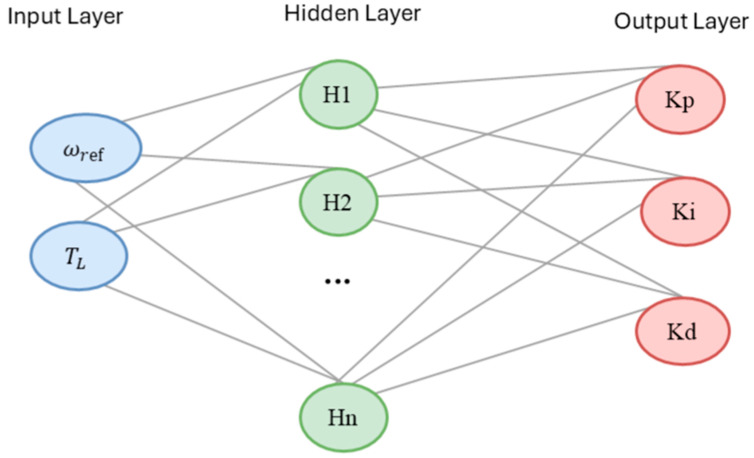
Architecture of ANN.

The Artificial Neural Network (ANN) serves as the core intelligence of the proposed controller, responsible for mapping the non-linear relationship between the operating conditions and the optimal control parameters. In this study, a multi-layer feed-forward network structure, specifically a Multi-Layer Perceptron (MLP), was utilized due to its universal approximation capability. The proposed ANN architecture consists of three distinct layers that are illustrated in Figure 2.

1. Input Layer: Comprises 2 neurons receiving the instantaneous system states: Reference Speed $\omega_{ref}$ and Load Torque $T_{L}$.
2. Hidden Layer: Contains a dynamic number of neurons determined by the optimization algorithm. To handle non-linearity, the hyperbolic tangent sigmoid activation function (σ) is employed
  (12) $$f\left(x\right)=\tanh\left(x\right)$$
3. Output Layer: Consists of 3 neurons that generate the optimal PID gains ($K_{P}$, $K_{i}$, $K_{d}$). A linear activation function is used in this layer to allow the outputs to take any real value required by the controller. The mathematical expression for the output of the k-th neuron in the output layer is given by:
  (13) $$y_{k}=\sum {\left(j=1\right)}^{\left(N_{h}\right)}w_{\left(jk\right)}^{\left(out\right)}\cdot \sigma \left(\sum {\left(i=1\right)}^{\left(N_{\left(in\right)}\right)}w_{\left(ij\right)}^{\left(hidden\right)}x_{i}+b_{j}\right)+b_{k}^{\left(out\right)}$$

The dataset of 100,000 samples was partitioned using the standard hold-out method: 70% for training, 15% for validation, and 15% for testing. The network consists of a single hidden layer, with the number of neurons and the learning rate determined by BKA optimization as described in Section 2.6. Training was performed using the Levenberg–Marquardt backpropagation algorithm with a maximum of 1000 epochs.

### 2.6. Proposed Method

An artificial neural network’s structural hyperparameters have a significant impact on its performance. While an incorrect learning rate might result in unstable convergence or stagnation, a suboptimal number of hidden neurons can induce either overfitting (excessive complexity) or underfitting (insufficient learning capacity). This paper suggests a BKA-Tuned ANN framework to overcome the drawbacks of human trial-and-error tuning.

In the proposed architecture, the Black Kite Algorithm is employed as a wrapper-based optimizer to automatically determine the optimal values of two critical hyperparameters:

Number of Hidden Neurons$\;(N_{h})$: Search range was set to [1, 50] to allow flexible complexity.

Learning Rate Search (η) range was set to [0.001, 0.1] to ensure stable weight updates.

**Figure 3:**
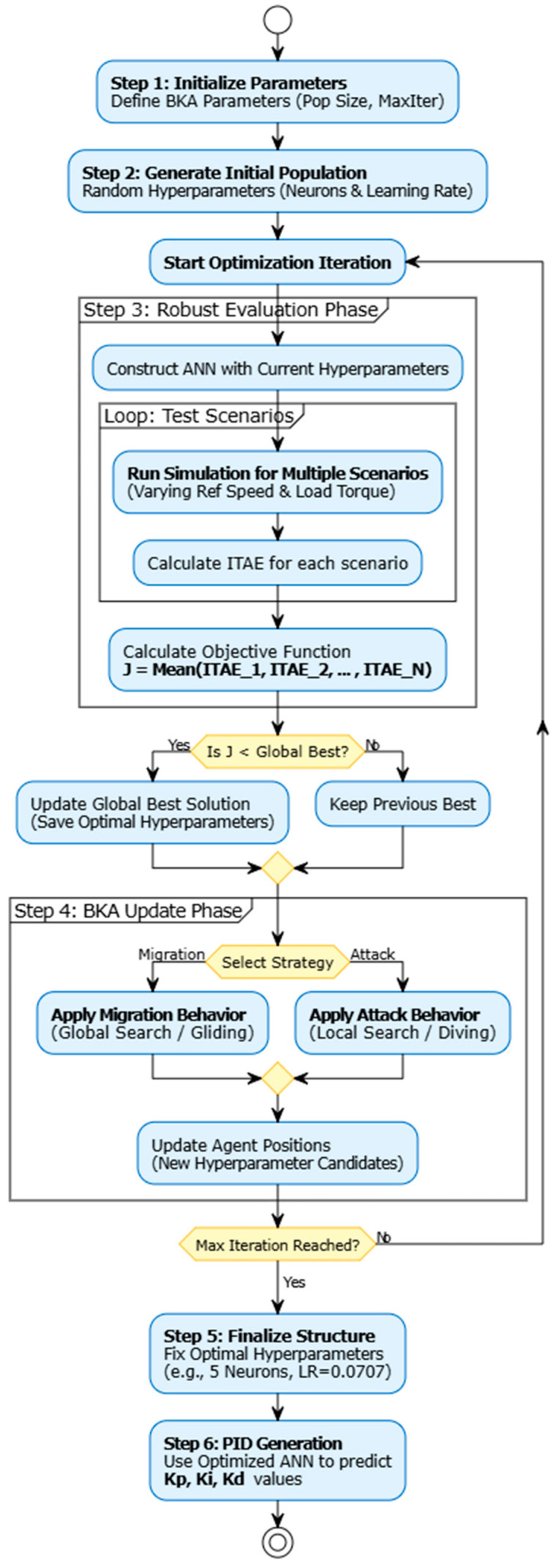
Flowchart of the proposed method.

The optimization process follows the flowchart illustrated in Figure 3. Each individual kite in the BKA population represents a candidate configuration vector $X_{i}=\left[N_{h},\eta \right]$. For every iteration, a temporary ANN is constructed and trained using the parameters defined by the kite. The fitness function (*F*) is defined as the Mean Squared Error (MSE) on the validation set:

(14) $$F\left(X_{i}\right)=1/M\sum {\left(j=1\right)}^{M}{\left(T_{j}-Y_{j}\right)}^{2}$$

where $T_{j}$ represents the target PID gains obtained from the dataset, $Y_{j}$ is the network’s prediction, and M is the number of validation samples. The BKA iteratively updates the population using the attacking and migration mechanisms described in Section 2.5 until the global minimum MSE is achieved. The final optimized network is then deployed for the online speed control of the DC motor. The theoretical basis for BKA’s ability to avoid local minima lies in its migration phase, which employs Cauchy-distributed random steps (Equation (7)). Unlike Gaussian distributions, the heavy-tailed nature of the Cauchy distribution enables large random jumps in the search space, allowing the algorithm to escape local optima that would otherwise trap gradient-based methods such as backpropagation. This property provides a theoretical convergence advantage over standard training techniques in non-convex optimization landscapes typical of ANN weight configuration.

## 3. Results and Discussion

All simulations, dataset generation, and optimization procedures were conducted using the MATLAB/Simulink R2022 environment running on a high-performance workstation equipped with an Intel(R) Core (TM) i9-14900HX (2.20 GHz) and 32 GB of RAM. The dynamic model of the DC motor was simulated using the Runge–Kutta (ode45) solver with a fixed step size of $10^{-4}$ s to ensure high-fidelity transient analysis. To train the BKA-tuned ANN, the generated large-scale dataset $\left(N=100,000\right)$ was randomly partitioned using the standard hold-out method: 70% for training, 15% for validation, and 15% for testing. The training process utilized the Levenberg–Marquardt (LM) backpropagation algorithm due to its fast convergence properties. The BKA optimization was executed with a population size of 30 and a maximum iteration count of 100. The BKA optimization was executed over multiple independent runs to account for the stochastic nature of the algorithm, and the best-performing configuration was selected for final evaluation. The results were found to be consistent across runs, with the optimal hyperparameters converging to the same compact network structure in most trials.

The comparative algorithms (hASO-SA, HHO, OBL/HGSO, ASO, HGSO, SFS, GWO, and SCA) were not re-implemented in this study. Instead, their reported PID parameters and corresponding performance metrics were directly adopted from the original publications, all of which employ the identical DC motor model used in this work.

The optimization process was executed to determine the most efficient ANN architecture for the BKA-based PID tuner. The search space for the number of hidden neurons was defined between [5, 50], and the learning rate between [0.001, 0.1].

As presented in Table 3, the Black Kite Algorithm converged to a compact network structure with 5 hidden neurons and a learning rate of 0.0707. The fact that the optimal neuron count converged to the lower bound indicates that the relationship between the operating conditions and PID parameters is smooth enough to be modeled with a low-complexity network. This compact structure is highly advantageous for real-time applications as it significantly reduces the computational burden on the microcontroller.

To evaluate the effectiveness of the proposed BKA-Tuned ANN, the optimal PID parameters obtained were compared against seven other well-known metaheuristic algorithms and control methods reported in the literature, including Hybrid Atom Search (hASO-SA), Harris Hawks Optimization (HHO), and Grey Wolf Optimizer (GWO).

As detailed in Table 6, the proposed BKA-ANN converged to a distinct set of control gains. Notably, the proposed method identified a higher proportional and derivative gain compared to traditional optimizers like GWO and SCA. This higher gain configuration enables the controller to react more vigorously to instantaneous errors, resulting in the significantly reduced rise time and settling time observed in the transient analysis.

Following the determination of the optimal control parameters listed in Table 4, a comprehensive simulation study was conducted to evaluate the time–domain performance of the proposed BKA-ANN controller. The system was subjected to a unit step reference speed input under no-load conditions to analyze its transient characteristics.

**Figure 4:**
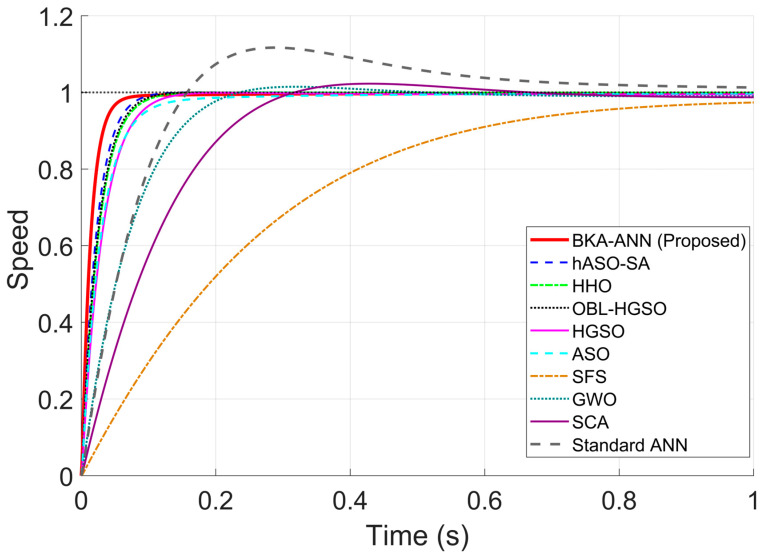
Step responses of DC motor with different control algorithms.

The comparative speed response of the DC motor controlled by the proposed method and other meta-heuristic algorithms is illustrated in Figure 4. The quantitative performance comparison of the proposed BKA-ANN controller against the Standard ANN and other metaheuristic optimization algorithms is detailed in Table 5.

When Figure 4 and Table 5 are examined, the results indicate that BKA-ANN achieves favorable performance in terms of dynamic response. The proposed solution achieved a rise time of 0.0303 s, which is roughly 35.5% faster than the second-best algorithm, hASO-SA (0.0470 s), and notably faster than GWO (0.1366 s) and SCA (0.2019 s). Moreover, BKA-ANN achieved stabilization at the reference speed in 0.0598 s with zero overshoot. On the other hand, hASO-SA, despite achieving near-zero overshoot, exhibited longer rise and settling times compared to the proposed approach. These results demonstrate the effect of BKA-based hyperparameter optimization on ANN training performance. The unoptimized Standard ANN produced a settling time of 0.7862 s and an overshoot of 11.6953%, confirming that backpropagation-based training without metaheuristic optimization leads to suboptimal gain configurations. This performance advantage can be attributed to BKA’s optimized hyperparameter configuration. The higher Kp and Kd values identified by BKA-ANN enable a more aggressive yet stable response to instantaneous errors. In contrast, algorithms such as GWO and SCA, whose population diversity mechanisms are prone to premature convergence, identified lower gain configurations resulting in slower dynamic responses and residual overshoots.

### 3.1. Frequency Analysis

**Figure 5:**
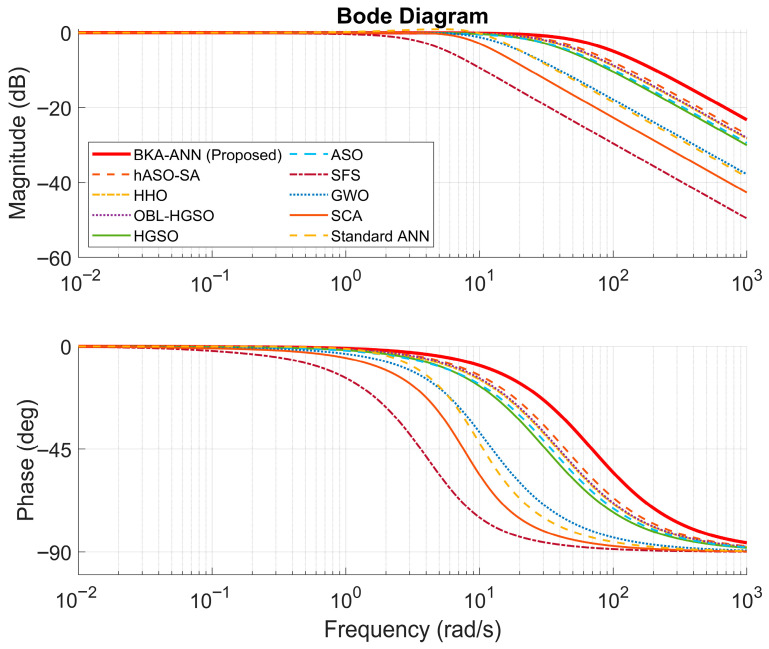
Comparison of the Bode Diagram with Different control algorithms.

The Bode diagrams of the proposed BKA-ANN and other comparative controllers are illustrated in Figure 5. In addition, the corresponding performance metrics (Phase Margin, Gain Margin, and Bandwidth) are shown in Table 6. An analysis of these measurements indicates that the BKA-ANN controller achieved the highest system bandwidth of 68.1203 rad/s, approximately 54% greater than hASO-SA (44.1802 rad/s), and notably higher than SFS, which yielded a bandwidth of 4.1183 rad/s. This higher bandwidth is consistent with the faster rise time observed in the transient response analysis. Additionally, all controllers maintained an unlimited gain margin, while the proposed approach achieved a phase margin of 90.52 degrees, which is consistent with the zero overshoot observed in the time–domain results. The Standard ANN obtained the lowest phase margin (73.96 degrees) and the smallest bandwidth (16.0104 rad/s), which is consistent with the higher overshoots and settling times observed in the transient analysis.

### 3.2. Robustness Analysis

To keep the system response within reasonable bounds, a robust controller is needed. In order to ascertain the stability of the suggested system in the event of uncertainties, robustness analysis was carried out. To do this, the torque constant $K_{t}$ and armature resistance $R_{a}$ of the DC motor were independently changed by ±25% and ±20%, respectively, to study the behavior of the system. Four possible operational scenarios were examined, and all scenarios are shown in Table 7.

**Figure 6:**
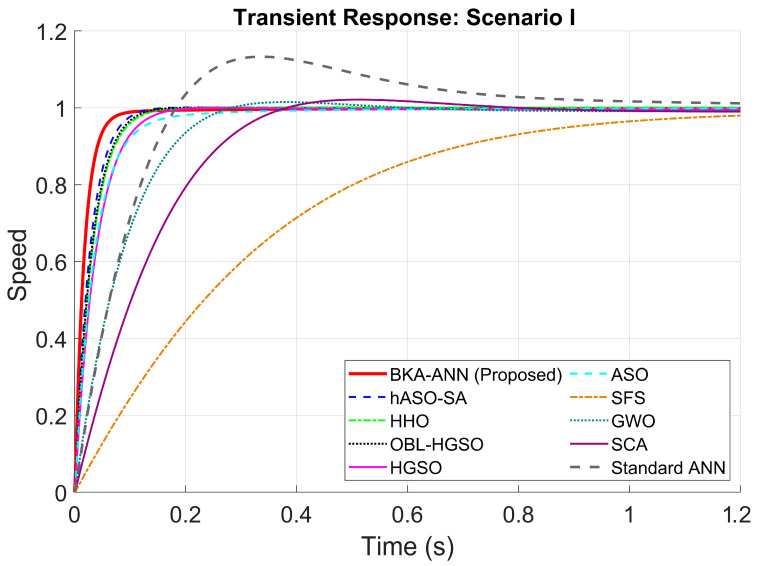
Step responses of DC motor for Scenario I.

**Figure 7:**
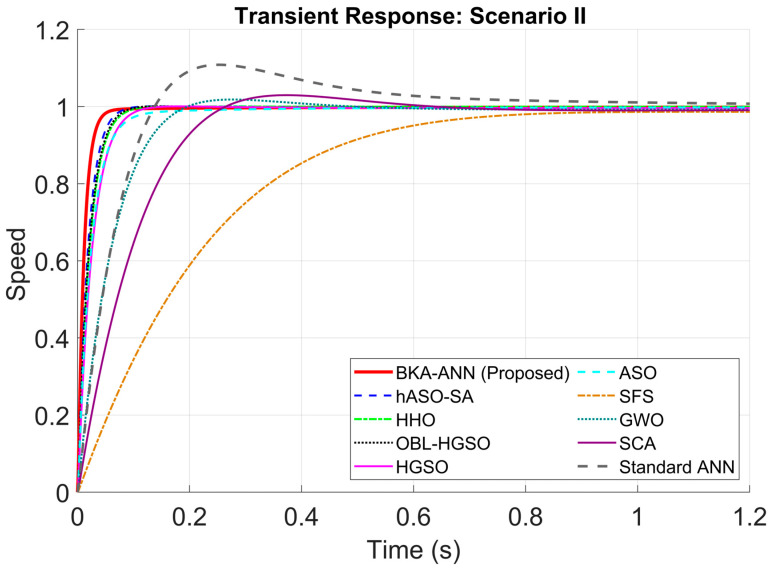
Step responses of DC motor for Scenario II.

**Figure 8:**
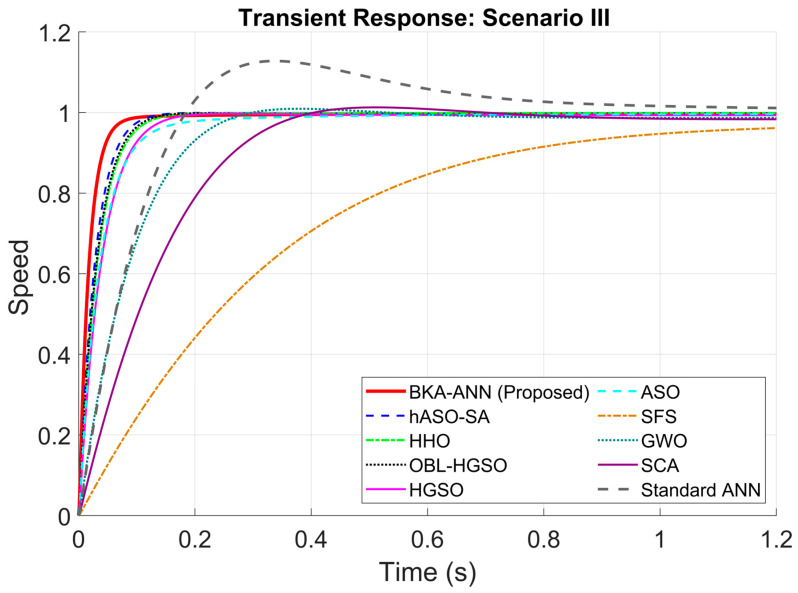
Step responses of DC motor for Scenario III.

**Figure 9:**
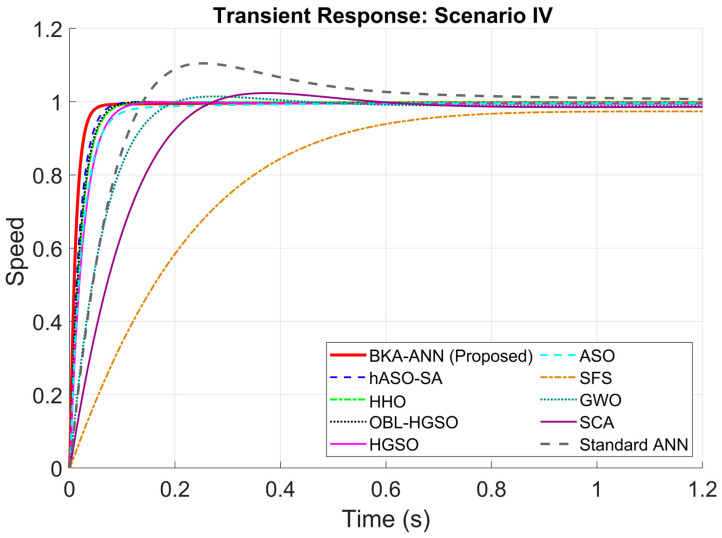
Step responses of DC motor for Scenario IV.

According to the scenario in Table 7, the results of the robustness analysis are presented in Table 8, Table 9, Table 10 and Table 11 and Figure 6, Figure 7, Figure 8 and Figure 9.

When Table 8 and Figure 6 are examined for Scenario I, it is seen that the proposed BKA-ANN controller is superior to all other methods in terms of transient response. Regarding the rise time which determines the system’s speed, BKA-ANN provided the fastest response with 0.0386 s, offering a significant speed advantage compared to its closest competitor, the hASO-SA method (0.0590 s). Similarly, the settling time, which represents the system’s stabilization speed, was measured as 0.0770 s, a value much lower than other hybrid algorithms (HHO, OBL/HGSO) and standard meta-heuristic methods (GWO, SCA).

When Table 9 and Figure 7 are examined for Scenario II, the proposed controller in this case attained an ultra-fast rise time of 0.0247 s, which is roughly 36% faster than hASO-SA, the second-best technique (0.0387 s). Also, the settling time of the BKA-ANN is 0.0770 s, which is far better than metaheuristic approaches like SCA (0.5562 s) and far quicker than the closest competitor, hASO-SA (0.1038 s). At the maximum overshoot value, one of the most critical parameters of control performance, BKA-ANN demonstrated perfect damping with a value of 0.00%.

When Table 10 and Figure 8 were examined for Scenario III, BKA-ANN achieved a rapid rise time of 0.0388 s and a settling time of 0.0790 s, significantly outperforming its closest competitor, hASO-SA. Lastly, when Table 11 and Figure 9 are examined for Scenario IV, while methods like SCA and GWO constantly produced oscillations between 1.5% and 3%, the Black Kite approach successfully tuned the ANN to handle complicated dynamics with minimal overshoot, thus explicitly validating the need for the approach.

Across all four robustness scenarios, BKA-ANN consistently achieved the fastest rise time and settling time while maintaining zero overshoot. This consistent performance can be attributed to two complementary factors. First, the ANN was trained on a large-scale dataset covering a wide range of operating conditions, enabling it to generalize beyond nominal parameters and produce appropriate PID gains even when motor parameters deviate significantly. Second, BKA’s Cauchy mutation-based migration phase ensured globally optimized hyperparameters, resulting in higher Kp and Kd values that provide aggressive yet stable error correction. In contrast, GWO and SCA produced overshoots ranging from 0.93% to 2.95% across scenarios due to their lower gain configurations caused by premature convergence. SFS exhibited the slowest settling times, reaching up to 3.7214 s in Scenario III, reflecting its limited step-size adaptability. The Standard ANN consistently produced the highest overshoots (10.46% to 13.30%) across all scenarios, confirming that unoptimized backpropagation training leads to suboptimal gain configurations. Among the competitive algorithms, hASO-SA, HHO, and OBL/HGSO maintained zero overshoot across scenarios; however, their rise and settling times remained substantially higher than those of BKA-ANN, suggesting that the exploration-exploitation balance of these algorithms results in suboptimal gain selections under parameter uncertainty conditions.

### 3.3. Load Disturbance Rejection Analysis

**Figure 10:**
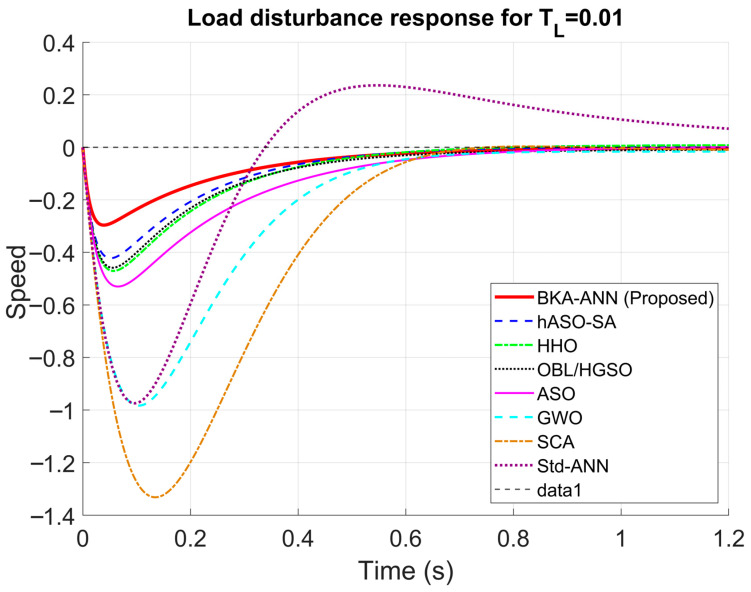
Step responses of DC motor for load disturbance.

The resilience of the proposed BKA-ANN controller to external load disturbances was meticulously assessed by introducing an abrupt step load torque of $T_{L}=0.01$ Nm, as depicted in Figure 10. A comparative study of the speed response trajectories demonstrates that the proposed technique displays enhanced stiffness, evidenced by a minimal undershoot of merely 0.2963 s, while the SCA algorithm had a considerable deviation of 1.3310 s. Moreover, in contrast to the standard ANN, which exhibited oscillatory behavior and overshoot, the BKA-ANN exhibited a smooth and asymptotic steady state without fluctuations. This result clearly demonstrates that the BKA-based tuning strategy guarantees superior disturbance rejection capacity, preserving system stability even during abrupt load disturbances, while alternative metaheuristic and unoptimized neural approaches fail to deliver a robust response. As reported in the literature, OBL/HGSO-PID also demonstrated improved disturbance suppression compared to HGSO, ASO, SFS, GWO, and SCA controllers under the same load torque condition [47]. Nevertheless, the proposed BKA-ANN achieved a notably lower undershoot of 0.2963 s compared to SCA (1.3310 s), while also outperforming OBL/HGSO in terms of settling time, indicating that BKA-based hyperparameter optimization yields a gain configuration with superior damping characteristics. The oscillatory behavior and overshoot observed in the Standard ANN further confirm that unoptimized backpropagation training yields gain configurations that lack adequate damping under sudden external disturbance.

### 3.4. Comparison of Energy and Maximum Control Signal of Controllers

**Figure 11:**
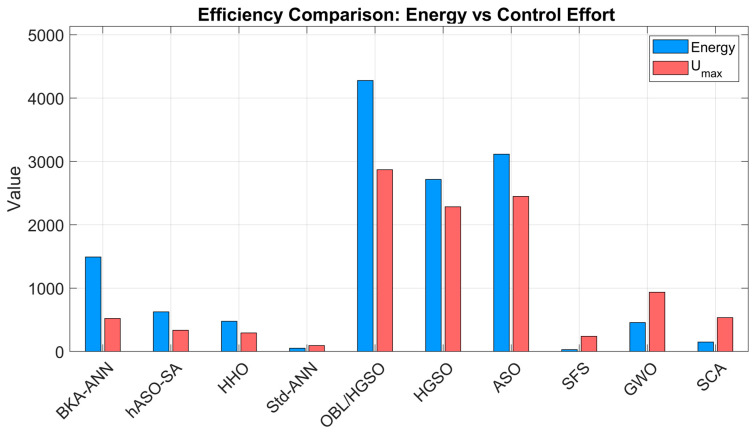
Comparison of Energy and the maximum control signal of the controller signals.

The control effort and energy consumption necessary for the controllers are essential considerations for realistic hardware implementation, as assessed in Table 12 and illustrated in Figure 11. Conventional algorithms such as SFS, GWO, and SCA demonstrate reduced overall energy consumption and maximum control signals; however, prior transient and robustness analyses have shown that this perceived efficiency comes at a high cost, including sluggish responses, excessive overshoots, and inadequate disturbance rejection. The suggested BKA-ANN demonstrates greater efficiency when compared to high-performance competitors with similar high-speed features. The BKA-ANN significantly restricts the maximum control effort to 521.3469, indicating an estimated 81% decrease relative to the OBL/HGSO-PID (2868.1327) and ASO-PID (2447.7437). Similarly, the suggested method’s total energy consumption (1492.5137) is approximately 65% less than OBL/HGSO’s (4277.5070). The significantly lower control effort and energy consumption of BKA-ANN compared to OBL/HGSO and ASO can be explained by the compact ANN architecture identified through BKA optimization. With only 5 hidden neurons, the network produces smooth and well-regulated PID gain outputs that avoid the aggressive voltage and current spikes associated with high-gain metaheuristic controllers. While OBL/HGSO and ASO identify high Kp and Ki values that drive fast responses, these configurations demand excessive control effort, making them less suitable for hardware implementation. BKA-ANN achieves a favorable balance between dynamic performance and energy efficiency, which is a critical requirement for real-world DC motor drive applications.

## 4. Conclusions

In this study, an Artificial Neural Network’s (ANN) hyperparameters were optimized using the Black Kite Algorithm (BKA). The PID controller parameters for DC motor speed regulation were then estimated using the resulting BKA-ANN model. The suggested BKA-ANN approach achieves competitive performance in DC motor speed control with zero overshoot and low settling times under all tested conditions, according to the simulation results and comparative analysis against nine algorithms, including hASO-SA, HHO, OBL/HGSO, ASO, HGSO, SFS, GWO, SCA, and the Standard ANN.

The proposed controller also demonstrated robustness under parameter uncertainty conditions. Even under four extreme uncertainty conditions involving simultaneous and large deviations in armature resistance and torque constant, as well as sudden external load torque disturbances, the system maintained full stability and performance integrity. The proposed approach’s efficacy was also assessed using frequency–domain analysis that included system bandwidth and phase margin. In comparison to metaheuristic controllers, BKA-ANN strikes a favorable compromise between dynamic performance and energy efficiency, according to the control effort and energy consumption analysis. Future research will focus on the real-time experimental validation of this design on hardware-in-the-loop platforms.

## Figures and Tables

**Table 1:** Parameters of the DC motor.

Parameter Description	Symbol	Value	Unit
Armature Resistance	$R_{a}$	0.4	Ω
Armature Inductance	$L_{a}$	2.7	H
Moment of Inertia	*J*	0.0004	kg⋅m^2^
Viscous Friction Coefficient	*B*	0.0022	N⋅m⋅s/rad
Torque Constant	$K_{t}$	0.015	N⋅m/A
Back EMF Constant	$K_{b}$	0.05	V⋅s/rad

**Table 2:** Flowchart of BKA [18].

1.Initialization	Population size (*N*), Max iterations (*T*), Bounds (*LB*, *UB*) Initialize random population $X_{i}$ and evaluate fitness $F_{i}$ Identify the global best solution: $X_{best}$
2.Main Loop	While (a< T) do:
3. Update Params	Update time-varying coefficients *n* and m
4. Attacking Phase	Generate random r∈[0,1]. if r > p $X_{new}=X_{i}\left(a\right)+\;n\;\cdot \left(1\;+\;sin\left(r\right)\right)\cdot \;X_{i}\left(a\right)\;$ else $X_{new}=X_{i}\left(a\right)+\;n\;\cdot \left(2r\;-\;1\right)\cdot \;X_{i}\left(a\right)$
5. Migration phase	Select random kite *k*, get fitness $F_{ri}.$ Generate C(0,1). if $F_{i}<\;F_{ri}$ $X_{new}=\;X_{new}+\;C\left(0,1\right)\cdot \left(X_{new}-\;X_{best}\right)$ else $\;X_{new}=\;X_{new}+\;C\left(0,1\right)\cdot \left(X_{best}-\;m.X_{new}\right)$
6. Boundary Check	Ensure $X_{new}$ is within [LB,UB].
7. Selection	Calculate fitness $F_{new}$ of $X_{new}$ if $F_{new}<\;F_{i}$ then $X_{i}=X_{new}$
8. Update Best	if any $X_{i}$ is better than $X_{best},$ update $X_{best}$
9. Termination	a=a+1. End while Output: Optimal solution $X_{best}$

**Table 3:** Optimal hyperparameters of the ANN found by BK.

Parameter Description	Lower Bound (LB)	Upper Bound (UB)	Optimal Value (Found)
Number of Hidden Neurons	5	50	5
Learning Rate	0.001	0.1	0.0707
Maximum Epochs	-	-	1000
Activation Function (Output)	-	-	Sigmoid
Activation Function (Output)	-	-	Linear

**Table 4:** Optimized PID controller parameters using different algorithms.

Controller/Method	$K_{p}$	$K_{i}$	$K_{d}$
BKA-ANN (Proposed)	24.9369	2.9429	4.9641
hASO-SA [46]	18.4258	3.3082	3.1755
HHO [48]	15.8581	3.6963	2.7732
OBL/HGSO [47]	16.9327	0.9508	2.8512
ASO [49]	11.9437	2.0521	2.4358
GWO [51]	6.8984	0.5626	0.9293
SCA [52]	4.5012	0.5260	0.5302
HGSO [47]	13.4430	1.2059	2.2707
SFS [50]	1.6315	0.2798	0.2395
Standard ANN	8.4521	12.2356	0.8541

**Table 5:** Transient responses of the DC motor with different control algorithms.

Controller/Method	Settling Time (s)	Rise Time (s)	Overshoot (%)
BKA-ANN (Proposed)	0.0598	0.0303	0.00
hASO-SA [46]	0.0866	0.0494	0.00
HHO [48]	0.1004	0.0568	0.00
SFS [50]	1.4709	0.5423	0.00
OBL/HGSO [47]	0.0951	0.0546	0.00
HGSO [47]	0.1158	0.0660	0.00
ASO [49]	0.1535	0.0692	0.00
GWO [51]	0.2052	0.1388	1.5062
SCA [52]	0.4899	0.2038	2.3056
ANN	0.7862	0.1141	11.6953

**Table 6:** Results of frequency response analysis with different controller algorithms.

Controller/Method	PM (deg)	GM (dB)	Bandwidth (rad/s)
BKA-ANN (Proposed)	90.52	∞	68.1203
hASO-SA [51]	89.80	∞	44.1802
HHO [48]	89.90	∞	38.5081
OBL/HGSO [52]	89.59	∞	39.8561
HGSO [52]	89.53	∞	31.7975
GWO [46]	84.02	∞	14.9018
SCA [47]	84.02	∞	14.9018
ANN	78.84	∞	10.1347
ASO [49]	91.24	∞	32.9113
SFS [50]	86.02	∞	4.1183

**Table 7:** Scenarios for robustness analysis.

Motor Parameter	Scenario I	Scenario II	Scenario III	Scenario IV
$K_{t}$	0.012	0.018	0.012	0.018
$R_{a}$	0.30	0.30	0.50	0.50

**Table 8:** Transient response performance comparison under Scenario I.

Controller/Method	Rise Time (s)	Settling Time (s)	Overshoot (%)
BKA-ANN (Proposed)	0.0386	0.0770	0.00
hASO-SA [51]	0.0590	0.1038	0.10
HHO [48]	0.0682	0.1206	0.12
OBL/HGSO [52]	0.0653	0.1136	0.03
ASO [49]	0.0846	0.1918	0.00
GWO [46]	0.1662	0.2455	1.50
SCA [47]	0.2429	0.5562	2.13
HGSO [52]	0.0824	0.1430	0.00
SFS [50]	0.6558	1.2050	0.00
Standard ANN	0.1344	0.9176	13.30

**Table 9:** Transient response performance comparison under Scenario II.

Controller/Method	Rise Time	Settling Time	Overshoot
BKA-ANN (Proposed)	0.0247	0.0479	0.00
hASO-SA [51]	0.0387	0.0688	0.00
HHO [48]	0.0448	0.0801	0.04
OBL/HGSO [52]	0.0431	0.0756	0.08
ASO [49]	0.0544	0.1167	0.00
GWO [46]	0.1152	0.1711	1.82
SCA [47]	0.1714	0.4752	2.95
HGSO [52]	0.0545	0.0954	0.00
SFS [50]	0.4437	0.8005	0.00
Standard ANN	0.0988	0.6999	10.83

**Table 10:** Transient response performance comparison under Scenario III.

Controller/Method	Rise Time	Settling Time	Overshoot
BKA-ANN (Proposed)	0.0388	0.0790	0.00
hASO-SA [51]	0.0594	0.1061	0.00
HHO [48]	0.0687	0.1238	0.00
OBL/HGSO [52]	0.0658	0.1163	0.00
ASO [49]	0.0854	0.2097	0.00
GWO [46]	0.1685	0.2531	0.93
SCA [47]	0.2474	0.3562	1.28
HGSO [52]	0.0831	0.1474	0.00
SFS [50]	0.7017	3.7214	0.00
Standard ANN	0.1354	0.9002	12.79

**Table 11:** Transient response performance comparison under Scenario IV.

Controller/Method	Rise Time	Settling Time	Overshoot
BKA-ANN (Proposed)	0.0248	0.0486	0.00
hASO-SA [51]	0.0389	0.0699	0.00
HHO [48]	0.0450	0.0815	0.00
OBL/HGSO [52]	0.0433	0.0768	0.00
ASO [49]	0.0547	0.1227	0.00
GWO [46]	0.1163	0.1747	1.43
SCA [47]	0.1736	0.4288	2.35
HGSO [52]	0.0549	0.0973	0.00
SFS [50]	0.4634	3.3544	0.00
Standard ANN	0.0994	0.6833	10.46

**Table 12:** Quantitative comparison of the controller performances based on energy and maximum control signals.

Controller/Method	Energy	$U_{max}$
BKA-ANN (Proposed)	1492.5137	521.3469
hASO-SA [51]	625.2594	335.9758
HHO [48]	476.1349	293.1781
Std-ANN	52.0328	93.8621
HGSO [52]	2714.9616	2284.143
OBL/HGSO [52]	4277.5070	2868.132
ASO [49]	3113.8590	2447.743
SFS [50]	30.6680	241.1315
GWO [46]	458.3019	936.1984
SCA [47]	150.2166	534.7012

## Data Availability

All produced data are available within the manuscript.
